# Effective Waterline Detection of Unmanned Surface Vehicles Based on Optical Images

**DOI:** 10.3390/s16101590

**Published:** 2016-09-27

**Authors:** Yangjie Wei, Yuwei Zhang

**Affiliations:** 1 College of Computer Science and Engineering, Northeastern University, Wenhua Str. 3, Shenyang 110819, China; yuweizhang@126.com; 2 State Key Laboratory of Synthetical Automation for Process Industries, Wenhua Str. 3, Shenyang 110819, China

**Keywords:** unmanned surface vehicle (USV), waterline detection, optical image blurring

## Abstract

Real-time and accurate detection of the sailing or water area will help realize unmanned surface vehicle (USV) systems. Although there are some methods for using optical images in USV-oriented environmental modeling, both the robustness and precision of these published waterline detection methods are comparatively low for a real USV system moving in a complicated environment. This paper proposes an efficient waterline detection method based on structure extraction and texture analysis with respect to optical images and presents a practical application to a USV system for validation. First, the basic principles of local binary patterns (LBPs) and gray level co-occurrence matrix (GLCM) were analyzed, and their advantages were integrated to calculate the texture information of river images. Then, structure extraction was introduced to preprocess the original river images so that the textures resulting from USV motion, wind, and illumination are removed. In the practical application, the waterlines of many images captured by the USV system moving along an inland river were detected with the proposed method, and the results were compared with those of edge detection and super pixel segmentation. The experimental results showed that the proposed algorithm is effective and robust. The average error of the proposed method was 1.84 pixels, and the mean square deviation was 4.57 pixels.

## 1. Introduction

Unmanned surface vehicles (USVs) eliminate risk and are a cost-saving tool for maritime applications in the surface zone. They provide unique capabilities and can be extensively used in many applications such as geophysical exploration, environmental monitoring, and water search-and-rescue operations [1,2,3]. However, realizing an autonomous USV system that can move in complicated surroundings such as a harbor, inland river, or flood disaster area is still an open challenge in the field of mobile robotics [4,5,6]. One of the main reasons is that sailing area detection in complicated and variant environments to provide a safe and feasible region for an autonomous USV system to operate is still not available.

The problem of detecting the sailing area of a USV system is actually equivalent to estimating the boundary line of the water area, which would help enable USV control and navigation. Nowadays, coastlines and shorelines can be detected with images captured by synthetic aperture radar (SAR) [7,8,9,10], which is typically mounted on a moving platform such as an aircraft or spacecraft. This has become a hot topic of research in marine geology because the field of view of a SAR system is quite large, and the images can be captured day or night, even in stormy weather and through clouds. However, because the resolution of SAR is comparatively low, SAR equipped on a USV system is not fit for precise modeling at short range. Therefore, it is difficult to use SAR for navigation and obstacle avoidance in an USV system.

Due to improvements in the precision and resolution of optical sensors, computer vision techniques have been used in many applications. Compared to other sensors, the requirements of optical sensors such as the experimental environment, operating cost, and equipment cost are all quite low [11,12,13]. Because of their advantages of direct and real-time imaging capability, optical technologies have been used to detect obstacles, achieve real-time vision feedback, and help improve the precision and efficiency of mobile robots. However, current waterline detection methods in the literature that are based on optical imaging are difficult to use in real USV systems. The reasons are as follows: (1) Optical waterline detection inspired by SAR-related techniques has problems with resolution and precision; as noted earlier, the imaging principles of SAR and optics are totally different, and the properties of their images are also different; for example, a SAR image consists of the traveling distance of an echo rather than intensity in an optical image. (2) The application area of current waterline detection methods based on optics is limited [14]; although several studies have considered waterline detection by using optical images, the sailing environments of the mobile robots are simple; for example, irregularly shaped reference objects may be required, or the shape of the entire waterline can be approximated by a straight line; when the system is in an unknown outdoor environment, it is difficult for these requirements to be fulfilled. (3) Some important factors such as illumination and shade have rarely been considered in current research on waterline detection with optical images, and the most widely used approach is threshold segmentation or edge detection of static images; when these techniques are applied to dynamic waterline detection by a USV system, the current precision, automation, and robustness are all low because of the complicated characteristics and details in an outdoor optical image. Many real and dynamic environmental factors should be researched for the development of an applicable waterline detection algorithm.

In order to solve these problems, we propose an efficient waterline detection based on structure extraction and texture analysis with respect to optical images. In contrast to current waterline detection methods based on SAR and optics, our method combines local binary patterns (LBPs) and the gray level co-occurrence matrix (GLCM) to evaluate the texture information of a dynamic image sequence when an UAV is moving on an outdoor water surface. Thus, our detection method considers the intensity contrast of neighboring pixels as well as the distance and orientation between them, so it is more comprehensive and robust than methods based only on the intensity or color of the water surface. Before detection, a preprocessing method based on structure extraction automatically removes noise textures resulting from wind, illumination, and motion. The main structures of the useful information remain. Our method does not involve any assumptions about the sailing environment of the USV and considers important factors such as the illumination, distance, and shade of the outdoor environment. Therefore, our method is a promising and robust approach to waterline detection by a USV.

The contents of this paper are organized as follows: firstly, in Section 2, the basic principles of structure extraction, LBPs, and GLCM are introduced, and their limitations and application areas are analyzed. Secondly, an efficient waterline detection based on structure extraction and texture analysis with respect to optical images is proposed in Section 3. Subsequently, in Section 4, the experimental results and error analysis based on the new method are given. Finally, Section 5 presents the conclusions of this paper.

## 2. Texture-Related Algorithms

Because of the complicated characteristics in an outdoor optical image capture by a USV system, the precision, automation, and robustness of current waterline detection methods are all low. The main challenge of waterline detection is to separate the bank area from the water area. However, there are many issues that can cause bad detection, such as illumination, reflections from land, waves, whirls, and presence of wakes from the USV or another vessel. Therefore, the properties of color, shapes and structures in an image are all required to be considered in any waterline detection algorithm. Because texture analysis includes both the intensity property of every pixel and the mutual relations among some pixels, texture is a promising property in waterline detection.

Texture is one of the fundamental features useful for describing image characteristics. Identifying the perceived qualities and properties of texture in images is an important step in image analysis, and there are many descriptors for texture description. Among them, the local binary patterns (LBPs) operator is one of the most widely used ones. Therefore, the LBPs operator is introduced and used first in our algorithm.

### 2.1. Local Binary Patterns for Texture Description

LBPs operator is a simple and efficient texture operator which labels the pixels of an image by thresholding the neighborhood of each pixel and considers the result as a binary number. Due to its discriminative power and computational simplicity, LBPs operator can be seen as a unifying approach to the traditionally divergent statistical and structural models of texture analysis. The basic idea for developing the LBPs operator was that two-dimensional surface textures can be described by two complementary measures: local spatial patterns and gray scale contrast.

Assume the local block is 3 × 3. LBPs operator can be denoted as:

(1) $${\mathrm{LBP}}_{P,R}(x_{c},y_{c})=\sum_{i=0}^{P-1}2^{i}H(g_{i}-g_{c})$$

(2) $$H(g_{i}-g_{c})=\left\{ \begin{array}{l} 1\;g_{i}-g_{c}\geq 0\\ 0\;g_{i}-g_{c}≺0 \end{array}\right.$$

where (*x_c_*, *y_c_*) are the coordinates of the center pixel, and its intensity is *g_c_*; *i* is the neighbor pixel of the center, and its intensity is *g_i_*; *R* is the radius of the neighborhood, and its unit is pixels; *P* is the number of pixels of a neighborhood whose radius is *R*. Because it is an 8-neighborhood calculation, the maximal value of the obtained LBPs description is 255, and it is an 8-bit image.

Global LBPs description over a large area of an image will be achieved in terms of local descriptions. Based on the idea of basic LBPs, many researchers have developed various texture operators, such as center-symmetric local binary pattern, dominant local binary patterns (DLBPs) [15], and completed LBPs (CLBPs) [16], and studied their applications towards texture based classification and segmentation, where a descriptor is required for texture extraction.

After calculation of LBPs description over an image, a descriptor is required to calculate the property of LBPs description. Current descriptors, such as histograms, normally used for texture classification and segmentation based on LBPs, cannot represent the occurrence frequency of certain ordinal relationships at different distances and orientations. However, in some cases, texture descriptions with different directions and orientations have some patterns for us to segment different regions of the image. Therefore, we need a new descriptor to calculate LBPs descriptions with variation of orientation and distance. Gray level co-occurrence matrix (GLCM) is a matrix defined over an image to calculate the distribution of co-occurring values at a given offset. It is a statistical approach for texture extraction in many fields and used alone or synergistically with other analysis to evaluate the images morphology. Therefore, According to the advantage of co-occurrence matrix framework [17], GLCM is then taken as the descriptor to statist the property of LBPs description in this paper.

### 2.2. Gray Level Co-Occurrence Matrix for Texture Extraction

The GLCM of an image is computed using a displacement vector *d*, defined by its radius *δ* and orientation *θ*. Three parameters together will be considered to describe an image through GLCM: the number of gray levels, the orientation angle and the length of displacement, and these parameters can be changed to improve the characterization. Mathematically, a co-occurrence matrix *C* is defined over an *n* × *m* image *I*, parameterized by an offset (Δ*x*, Δ*y*), as:

(3) $$C_{\Delta x,\Delta y}(i,j)=\sum_{p=1}^{n}\sum_{q=1}^{m}\left\{ \begin{array}{l} 1\;I(p,q)=i\;\mathrm{and}\;I(p+\Delta x,q+\Delta y)=j\\ 0\;\mathrm{otherwise} \end{array}\right.$$

where *I* and *j* are normally the image intensity values of the image, in this paper they are LBPs description of the pixel whose spatial positions are denoted by *p* and *q*. The offset (Δ*x*, Δ*y*) depends on the direction used *θ* and the distance at which the matrix is computed *d*. The value of the image originally referred to the grayscale value of the specified pixel, but could be anything, from a binary on/off value to 32-bit color and beyond. What we need to clarify is that the image we use in this paper is that obtained after LBPs calculation.

Then, the normalization equation whose formula follows is considered:

(4) $$P(i,j)=\frac{C_{\Delta x,\Delta y}(i,j)}{\sum_{i,j=1}^{n,m}C_{\Delta x,\Delta y}(i,j)}$$

where *P*(*i*, *j*) is the probability.

The properties of an image texture are indirectly extracted by using the co-occurrence matrix from image indicators, such as entropy indicator, mutual indictor, and energy indictor. The entropy indicator measures the disorder or complexity of an image texture and it can be denoted as:

(5) $$\mathrm{Entropy}=-\sum_{i,j=1}^{n,m}P(i,j)\log P(i,j)$$

The energy indictor measures the difference between the highest and the lowest values of a contiguous set of pixels, and it is strongly but inversely correlated to entropy:

(6) $$\mathrm{Energy}=\sqrt{\sum_{i,j=1}^{n,m}P{\left(i,j\right)}^{2}}$$

The mutual indictor supplies further information by which the uncertainty about one variable is reduced by the given knowledge of the second variable, and it is shown as:

(7) $$\mathrm{Mutual}\;\mathrm{information}=-\sum_{i,j=1}^{n,m}P(i,j)\log\left(\frac{P(i,j)}{P_{i(i)}P_{j(j)}}\right)$$

where $P_{i(i)}=\sum_{i=1}^{n}P(i,j),P_{j(j)}=\sum_{j=1}^{m}P(i,j)$.

GLCM gives information about patterns in LBPs descriptions over the optical image, and it is providing a way of extracting second-order statistical LBPs features. However, as is well known, this approach is sensitive to illumination variations. In our application, the application environment is outdoors and complicated, and our method has to consider the influence of illumination variation. Therefore, an autocorrelation method is required in a complicated application to assess the amount of regularity as well as the main structure of the texture presented in an image. In this paper, we choose the structure extraction proposed by Xu [18], which is simple yet effective to partially remove the influence of some environmental factors.

### 2.3. Structure Extraction

It is normal in real applications that some images share the similarity that semantically meaningful structures are blended with or formed by texture elements, and these images are called “structure + texture” images. Although human visual system is fully capable to understand these pictures without needing to remove textures, extract structures by a computer is much more challenging. Therefore, a method based on novel local variation measures to accomplish texture removal was proposed.

First, the total variation model that simply uses a quadratic penalty to enforce structural similarity between the input and output is expressed as:

(8) $$\arg\underset{S}{\min}\sum_{t}\left\{\frac{1}{2\lambda }{\left(S_{t}-I_{t}\right)}^{2}+\left|{\left(\nabla S\right)}_{t}\right|\right\}$$

where *I* is the input image, which could be the luminance (or log luminance) channel and *t* indexes 2D pixels. *S* is the resulting structure image. The data term (*S_t_* − *I_t_*)^2^ is to make the extracted structures similar to those in the input image. Σ*_t_*|(∇*S*)*_t_*| is the total variation (TV) regularizer, written as:

(9) $$\arg\underset{S}{\min}\sum_{t}\left\{\frac{1}{2\lambda }{\left(S_{t}-I_{t}\right)}^{2}+\left|{\left(\nabla S\right)}_{t}\right|\right\}$$

(10) $$\sum_{t}\left|{\left(\nabla S\right)}_{t}\right|=\sum \left|{\left(\partial_{x}S\right)}_{t}\right|+\left|{\left(\partial_{y}S\right)}_{t}\right|$$

with the anisotropic expression in 2D. $\partial_{x}$ and $\partial_{y}$ are the partial derivatives in two directions.

The improved model is written as:

(11) $$\arg\underset{S}{\min}\sum_{t}{\left(S_{t}-I_{t}\right)}^{2}+\lambda \cdot \left(\frac{D_{x}(t)}{L_{x}(t)+\varepsilon }+\frac{D_{y}(t)}{L_{y}(t)+\varepsilon }\right)$$

where:

(12) $$D_{x}(t)=\sum_{l\in R(t)}g_{t,l}\cdot \left|{\left(\partial_{x}S\right)}_{l}\right|,D_{y}(t)=\sum_{l\in R(t)}g_{t,l}\cdot \left|{\left(\partial_{y}S\right)}_{l}\right|$$

(13) $$L_{x}(t)=\left|\sum_{l\in R(t)}g_{t,l}\cdot {\left(\partial_{x}S\right)}_{l}\right|,\;L_{y}(t)=\left|\sum_{l\in R(t)}g_{t,l}\cdot {\left(\partial_{y}S\right)}_{l}\right|$$

where *l* belongs to *R*(*t*), the rectangular region centered at pixel *t*. *D_x_*(*t*) and *D_y_*(*t*) are windowed total variations in the *x* and *y* directions for pixel *t*, which count the absolute spatial difference within the window *R*(*t*). *g_t,l_* is a weighting function defined according to spatial affinity, expressed as:

(14) $$g_{t,l}\propto \exp\left(-\frac{{\left(x_{t}-x_{l}\right)}^{2}+{\left(y_{t}-y_{l}\right)}^{2}}{2\sigma^{2}}\right)$$

With Equation (14), a number of new applications to manipulate, render, and reuse the immense number of “structure with texture” images and drawings that were traditionally difficult can be edited properly. It is promising for preprocessing when there is a global noise texture in an image, especially when the image is captured during moving. Therefore, it is used as a preprocessing method to remove the global influence resulted from illumination or movement of the vehicle. In the following section, we will introduce our waterline detection algorithm based on structure extraction and texture analysis.

## 3. Waterline Detection Algorithm

In this paper, we propose an efficient waterline detection method based on structure extraction and texture analysis with respect to optical images and present a practical application to a USV system for validation. Our algorithm can be divided into three parts: image preprocessing, the first segmentation, and the final segmentation. In the following, we will introduce each of these procedures in detail.

### 3.1. Image Pre-Processing

**Figure 1:**
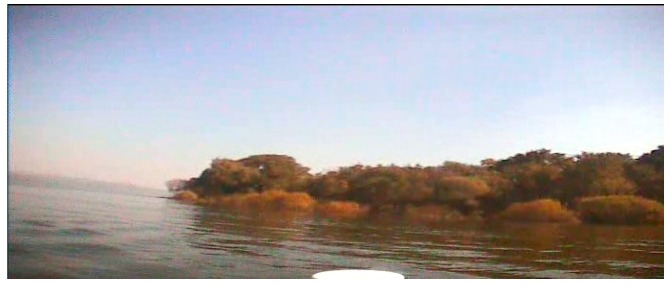
The image before structure extraction.

**Figure 2:**
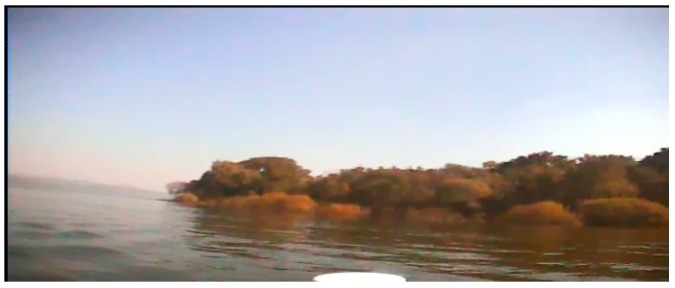
The image after structure extraction.

Compared to SAR images, optical images, especially color images, contain more detailed information. However, since our USV is presumed to be moving in a complicated outdoor environment when we capture the sample images, it is unavoidable that there will be some negative textures on the whole captured images due to the influence of illumination, USV motion, and wind disturbance. These are “structure + texture” images, and these noise textures could interfuse into our texture analysis in the next steps. Therefore, the first task of our detection method is to remove the unusual textures and remain the main structures. The operator we use in this paper is the inherent variation to extract the main structures, and the formula is shown as Equation (10). Figure 1 and Figure 2 are the images before and after our structure extraction. From them, we can see that through structure extraction, the sample image is smoother, and the noise textures those distribute evenly on the image are removed. At the same time the global structural features to separate each main structure have been remained.

**Figure 3:**
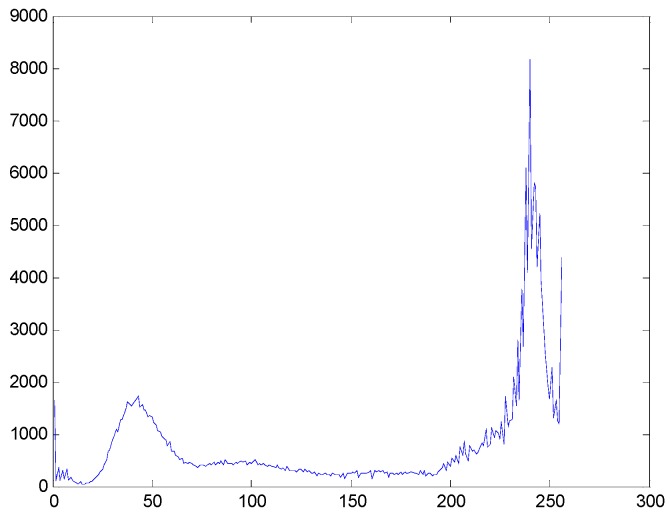
The histogram of the *B* signals in the entire image.

**Figure 4:**
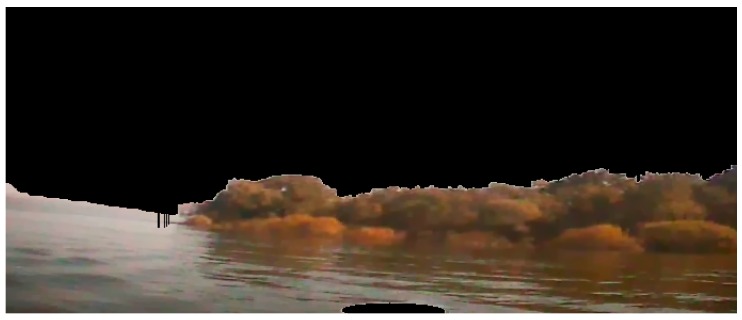
The result after our image preprocessing.

Then, in order to reduce the processing time and to improve the real-time ability of our method, it is necessary to segment the background (i.e., sky in our sample image) which almost has no textures and the intensity is distributed evenly. In this paper, we use the *B* value from the blue channel in the color image to segment the background, because the *B* signals of the background area are higher than other signals. Figure 3 is the histogram of the *B* channel, where we can see that there is an obvious peak when the intensity value is higher than 200. Based on the histogram analysis, we could segment the background area from the sample image, and the result can be seen in Figure 4.

### 3.2. The First Segmentation

**Figure 5:**
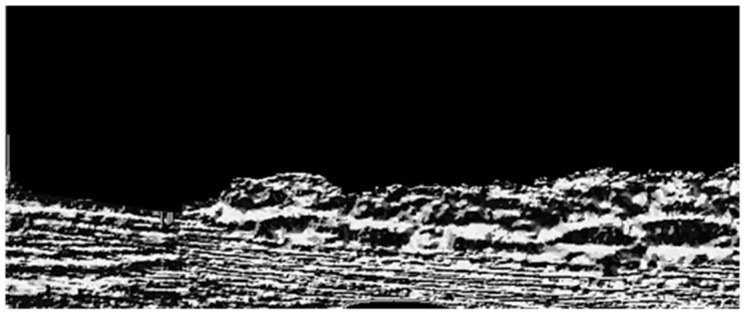
The result of LBP calculation based on our image preprocessing in Section 2.1.

From Figure 4, we can find that it is difficult to separate the water area from the proposed image with methods based on threshold segmentation, because their intensity values, such as *R*, *G*, and *B*, are very close due to illumination and shade. Since the water texture consists of comparatively regular shapes, in this paper, we calculate the texture of the preprocessed image show as Figure 4 with the LBPs operator, and each image block has 3 × 3 pixels. Figure 5 is our calculation result, where we can see that the texture of the trees on the bank is different from that of the water. According to the principle of LBPs, the obtained image is an eight bit gray image.

**Figure 6:**
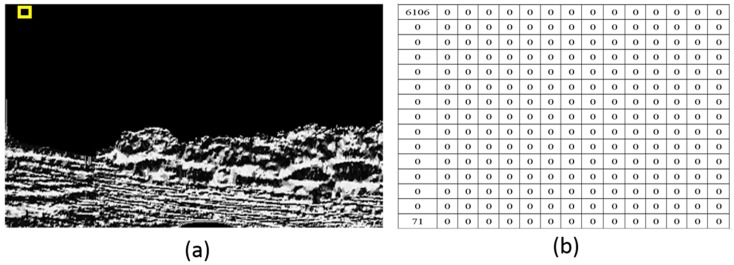
The GLCM when the center pixel is (15, 15). (**a**) The LBP image where the calculation block shown with the yellow rectangle is the calculation position; (**b**) The GLCM result.

**Figure 7:**
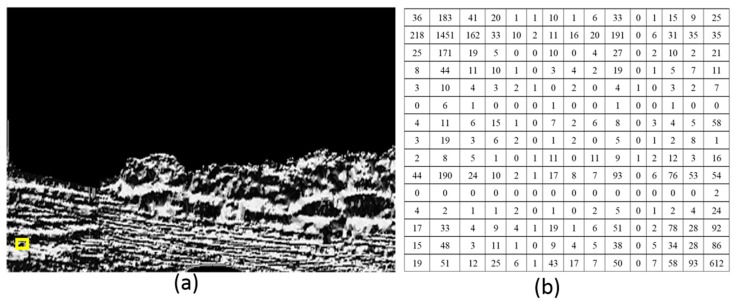
The GLCM when the center pixel is (275, 15). (**a**) The LBP image where the calculation block shown with the yellow rectangle is the calculation position; (**b**) The GLCM result.

Then, in order to segment the water area after calculation with the LBPs operator, we require an indicator to evaluate the texture variation and to separate two neighboring image blocks. As we mentioned in Section 2, GLCM is a statistical approach considering orientation and distance. Therefore, we use GLCM to estimate LBPs distribution. First, we divide the entire LBPs image into many image blocks and calculate the GLCM value of each LPBs image block. In this calculation, the radius *δ* is 4 and the orientation *θ* is 0°, 45°, 90°, and 135°. Figure 6 is the GLCM result when the center pixel is (15, 15) and the block size is 30 × 30. Figure 6a is the LBP image where the block shown with the yellow rectangle is the calculation position; Figure 6b is the calculated GLCM. Figure 7 is the GLCM when the center pixel is (270, 15) and the block size is 30 × 30. Figure 7a is the LBP image where the block shown with the yellow rectangle is the calculation position; Figure 7b is the calculated GLCM. From these figures, we can see that the GLCM is changing along the same column. The reason is that the texture property is changing. Subsequently, we choose the entropy indictor to evaluate the GLCM distribution of each image block. The evaluation method is to calculate the difference entropy between two neighboring image blocks along each column and to obtain the differential curve of this column. The highest difference of entropy value is found when the values of *P*(*i*, *j*) are allocated quite uniformly throughout the matrix. This happens when the image has no pairs of gray levels, with particular preference over others. When GLCM is calculated, the size of each image block may influence the scale of its estimation. Therefore, for optical images with different scenes, normally an optimal size needs to be obtained through practice, and an overlap between two neighboring blocks can be used to reduce the influence of the block size. The size of each image block in this paper is 30 × 30 pixels, and the overlap of two neighboring blocks is 10 pixels.

**Figure 8:**
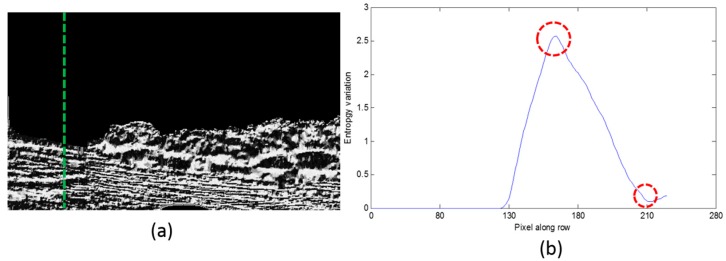
The entropy differential curve when two areas exist. (**a**) The LBP image where the calculation position is shown with the green dashed line; (**b**) The entropy differential curve.

**Figure 9:**
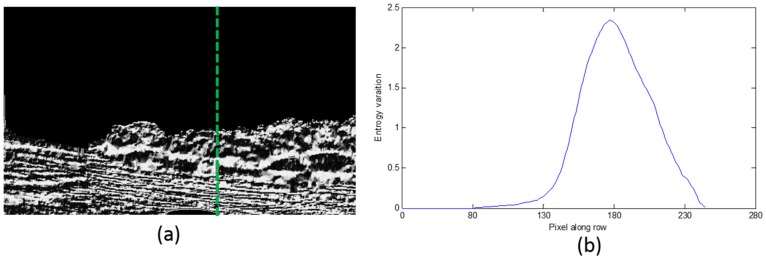
The entropy differential curve when three areas exist. (**a**) The LBP image where the calculation position is shown with the green dashed line; (**b**) The entropy differential curve.

The entropy differential curves along two typical columns are shown in Figure 8 and Figure 9. Figure 8a and Figure 9a are the LBP image where the calculation position is shown with the green dashed line; Figure 8b and Figure 9b are the entropy differential curves, where the horizontal axis is the number of difference entropy calculation along each column, and the calculation is from the top of the image to the bottom of the image. The vertical axis is the difference entropy between two neighboring image blocks. In Figure 8b, there are two different areas including the water area and the sky, so only one peak exists on the differential curve, while in Figure 9b, there are some trees between the water area and the sky, therefore we can find two bending points. These points are the separation lines of three different areas, and what we require to do is to detect the positions of the peaks and to define them as the separation positions of this column. The first separation position from bottom to top is what we desire, because the water area is the nearest to the camera.

**Figure 10:**
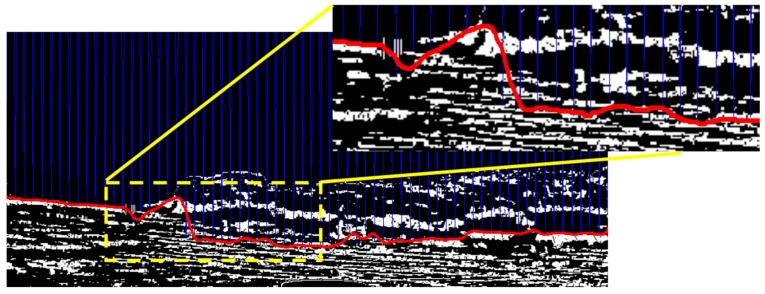
The result of the first segmentation.

Finally, based on the separation positions on each column, we could obtain the entire segmentation line of the sample image as shown in Figure 10, where the solid line along the horizontal axis is the segmentation line between the water area and other areas. The segmentation result in the area of the yellow dashed rectangle are zoomed and shown at the top-right corner of Figure 10. From this figure, we can see that the waterline is not very precise at some positions. The reason is that in order to decide the boundary of two types of textures we only compare the entropy values between neighboring image blocks along each column. That means we calculate the differential curves in the vertical direction. However, the GLCM variation of the image blocks in the horizontal direction has not been considered.

### 3.3. Final Segmentation

From Figure 10, we can find that after the first segmentation, the segmentation line between the water area and the bank is not very precise at some points. In order to improve the precision of our detection method, we add the final segmentation to consider the global properties of the image. In this step, if there are some unusual peaks appearing on the detected waterline of the first segmentation, we define a local searching area with a reference line, and search for the desired positions in the searching area to compensate the result of the first segmentation. The searching target that we desire has to fulfill two requirements: (1) it must be in a bright stripe area; (2) it is the nearest point to the reference line on the same row.

First, we need to define the local searching area. In this paper, we detect a short straight line as the reference line from the LBPs image. Because the texture of water is mostly straight stripes due to waves, rather than circular arcs of trees. It is reasonable to use Hough transformation to extract the straight line, which is the highest and longest, and to define it as the reference line. Then, the reference line is prolonged to intersect the entire image with its slope, and the local searching area is subsequently defined around the referenced line. If the coordinate of the *j*th row on the reference line is *i*, we define the searching area along the *j*th row is [*i* − Δ*a*, *i* + Δ*a*]. When we find some unusual peaks appearing on the detected waterline of the first segmentation, we start searching in this scope. Δ*a* can be defined in a real application, and its unit is pixel.

**Figure 11:**
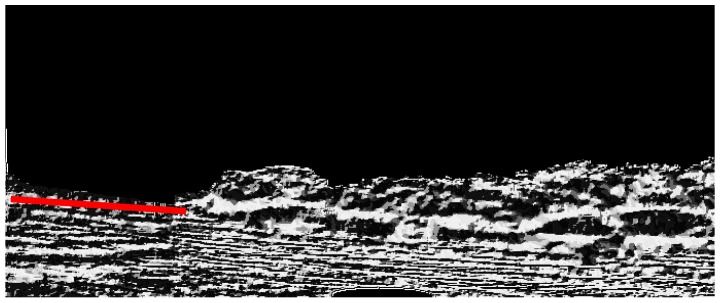
The reference line for local searching.

**Figure 12:**
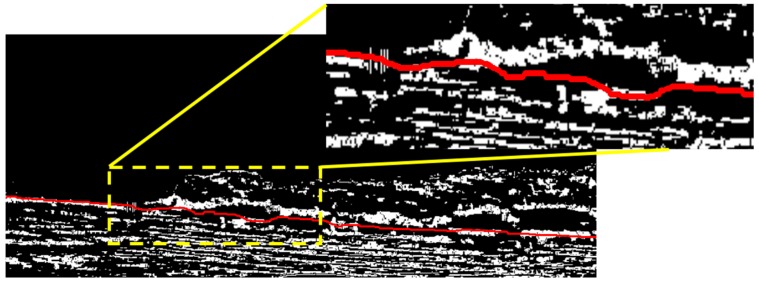
The result of the final segmentation.

The detection result of Hough transformation can be shown in Figure 11, and we can believe that the true waterline is around the Hough line because the shape of the separation line between the bank and the water area is changing slowly, and it is not reasonable to find some sudden peaks on the true waterline. Therefore, the Hough line and the local searching area can be used to compensate the detection result of the first segmentation when some unusual peaks exist on it, and the searching requirements have been noted earlier. The final segmentation result is shown in Figure 12, and the segmentation result in the area of the yellow dashed rectangle are zoomed and shown at the top-right corner of Figure 12, where the peaks from the first segmentation have been removed, and the detected waterline is smooth and close to the ground truth.

**Figure 13:**
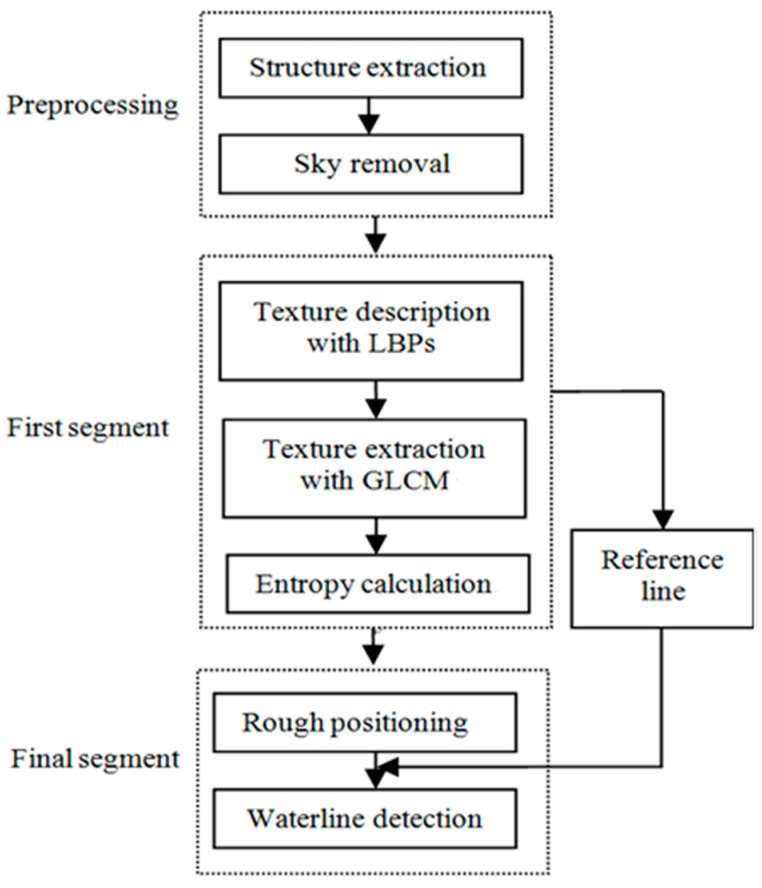
The flow of our detection algorithm.

Figure 13 is the flow of our entire algorithm, which can be divided into seven steps:

(1) Extract the main structures with Equation (10) and the input color image *I*, and the calculated image *S* is the result;
(2) List the histogram of the blue signals with *S*, and segment the sky with a threshold;
(3) Calculate LBPs of *S* with Equations (1) and (2), and attain *S*_LBPs_, which is an 8-bit image;
(4) Calculate GLCM of *S*_LBPs_ with Equations (3) and (4), each image block is 30 × 30 pixels, and attain *S*_GLCM_(*i*) of image block *i*;
(5) Calculate the entropy of each image block *S*_GLCM_(*i*). After scanning of an entire row, a differential curve of this row is obtained, and the last peak from top to bottom is the separation position;
(6) Detect the longest and highest straight line with *S*_LBPs_ using Hough transformation, and define the local searching area with Δ*a* = 20 pixels;
(7) Combine the result of Steps (5) and (6), the final waterline is obtained. The combination condition is that if a peak exists in the result of Step (5), a local search is conducted in the local searching area on *S*_LBPs_ until a desired point is found.

## 4. Experiments

**Figure 14:**
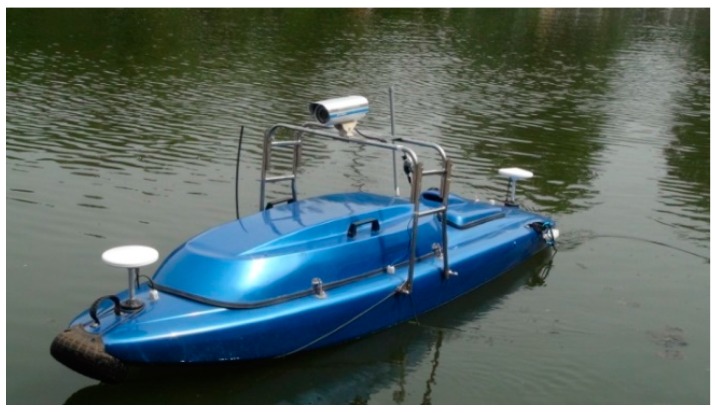
The USV system in our experiments.

In the practical application, the waterlines of many images captured by an USV system moving along an inland river are detected with the proposed method, and the results are compared with those of edge detection and super pixel segmentation. The USV system was designed by the Shenyang Institute of Automation (Shenyang, China), and it is shown in Figure 14. Its basic parameters are listed in Table 1.

The USV system is composed of four sub-systems, including on-board control computer sub-system, communication sub-system, sensor and perception sub-system, and the ground station sub-system. The sensor sub-system contains the GPS-INS system, which is used to localize the USV and obtain some inertial state, and the VS250DH vision sensor, developed by the MicroVision Company (Redmond, WA, USA). The detailed specifications of the vision sensor are listed in Table 2. The on-board control computer can be used to record the experimental data. The ground station in the application is used to tele-control the USV system to collect the required videos and images.

### 4.1. Experiment under Different Situations

In this section, four groups of different experiments with different conditions are conducted to verify the feasibility of the new proposed algorithm, and the experimental conditions are listed out in Table 3.

**Figure 15:**
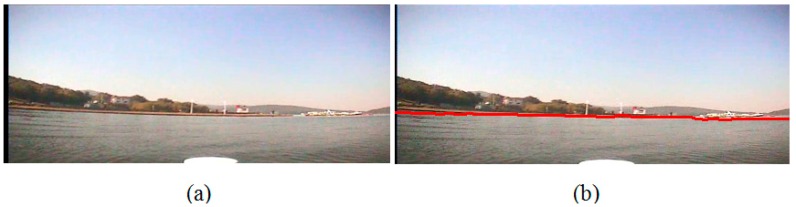
Waterline detection of a far bank. (**a**) The image before our detection; (**b**) The image after our detection.

In Experiment I, we conduct the experiment on the image which is captured at a sunny noon when the USV is far away from the bank. The sunlight is projected evenly on the surface of the water area, and there is an obvious riverbank between the water area and the trees on the bank. There is no shade of the trees on the water surface. Besides, because the bank is far away from the camera, the scene on the bank is very small in the sample image so that the detailed texture of the trees is not very clear. Figure 15a is the original image, and Figure 15b is the detection result with our waterline detection method. From them, we can see that the detected waterline is very precise. Furthermore, on the right side of the image there is a boat pulled up alongside the dock, and our method can separate it from the water area precisely too.

**Figure 16:**
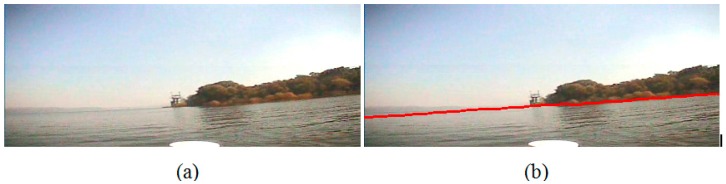
Waterline detection of a far bank and a distant mountain. (**a**) The image before our detection; (**b**) The image after our detection.

**Figure 17:**
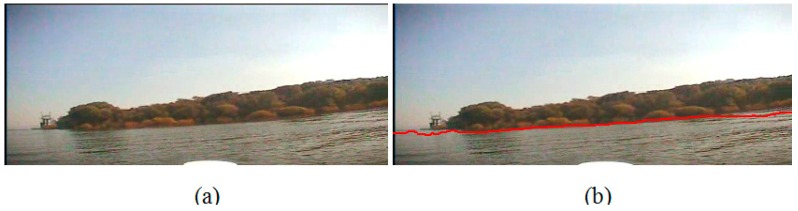
Waterline detection of a closer bank with uneven illumination. (**a**) The image before our detection; (**b**) The image after our detection.

Second, we conduct the experiment on the image which is captured when the USV system is closer to the bank than that in experiment I; however, there is no obvious line between the water area and the bank. The sun is on the top right corner of the image, where is brightest due to the uneven illumination, and on the water area the right side is brighter than the left side. Besides, in Figure 16, there is a mountain which is far away from the water, and the intensity of the mountain is close to that of the water area. Figure 16a and Figure 17a are the original images, and Figure 16b and Figure 17b are the detection results, where we can see that the detected waterlines are clear enough to separate the water area from the trees and the mountain, and the uneven illumination has not influenced the segmentation result.

**Figure 18:**
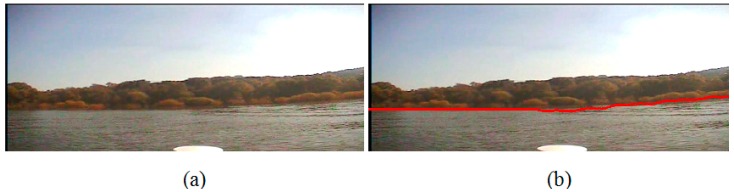
Waterline detection of a closer bank with uneven illumination. (**a**) The image before our detection; (**b**) The image after our detection.

In Experiment III, we capture the images under more complicated situdtion. The bank and the water area in the image are coser to the camera, so that the entire waterline is not as stright as those in the first two experiments. The left side of the water area is brighter than the right side because of the uneven illumination. Figure 18a,b is the original image and our detection result, respectively. From Figure 18 we can see that our algorithim can precially recongize the angle variation of the waterline, and the detected waterline is close to the true ground.

**Figure 19:**
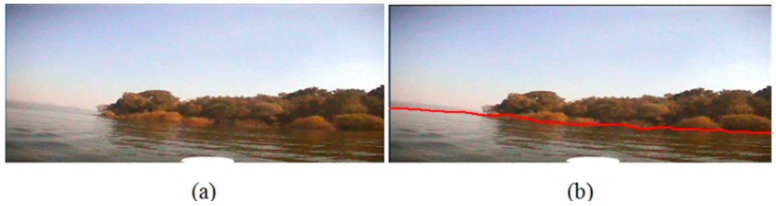
Waterline detection of a close bank with serious shade. (**a**) The image before our detection; (**b**) The image after our detection.

**Figure 20:**
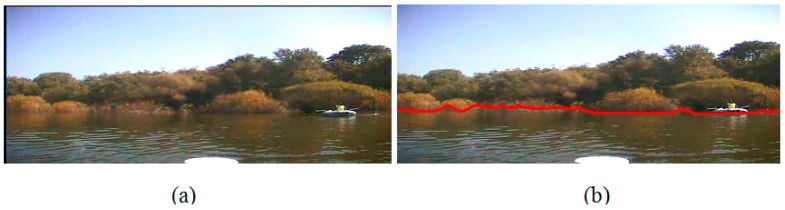
Waterline detection of a close bank with serious shade. (**a**) The image before our detection; (**b**) The image after our detection.

Finally, in Experiment IV, we capture the images those are full of shade, where the bank is so close to the water area that the shade of the trees projects on the water surface, and the colour of the water area is as same as that of the trees because of the illumination. Furthemore, since the USV is close enough to the bank when we capture the images, the waterline is far from a straight line. Figure 19b and Figure 20b are our detection result. From them we can see that our algorithim has not been influenced by the shade, and the detected lines can descirbe the variation of the water area, even when a boat is on the water surface. Based on all the experimental results, we can obtain the following conclusions:

(1) When the bank is far away from the USV system, the waterline is close to a straight line, and the scenes on the bank are small. Our algorithm can separate the small scenes on the bank even the color of the mountain is close to that of the sky;
(2) When the bank is closer to the USV, the angle of the waterline may be changing, rather than a straight line; the brightness of the entire image can be influenced by the uneven illumination. However, our algorithm can segment the water area from the image, and the distance and the brightness of tress and buildings on the bank have no influence on our method;
(3) For a very close image, the waterline is a complicated curve, and if the sun is behind the trees, the shade of the trees projects on the water surface. Our algorithm can detect the waterline, even when the color of the water area is close to that of the trees.

### 4.2. Error Analysis

**Figure 21:**
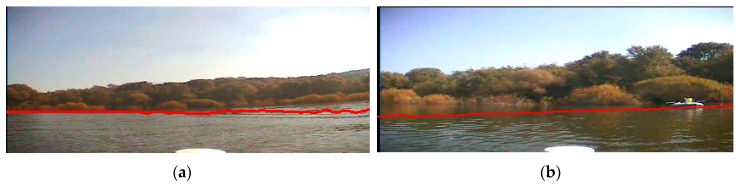
Waterline detection result without our preprocessing stage. (**a**) The detection result when there is a brightness layer; (**b**) The detection result when there is a shade.

First, we test the significance of our preprocessing stage in our waterline detection method. In this experiment, we do not use our preprocessing method, and all the parameters of the first segmentation and the final segmentation are as same as what we use in the experiments of Section A. The tested images those we use are Figure 18a and Figure 20a, and the result is shown in Figure 21, where we can see that without the preprocessing stage, our detection method is sensitive to waves and shades: when the wave results into a brightness layer, which is more obvious than others, the precision of our method has been reduced, as shown in Figure 21a; when the water surface is covered by a shade and the color of the shade is closer to that of the trees on the bank, the precision of our method has reduced, as shown in Figure 21b. Therefore, our preprocessing stage is necessary to reduce the sensitivity of our method. The red solid line is the line detected by our method.

**Figure 22:**
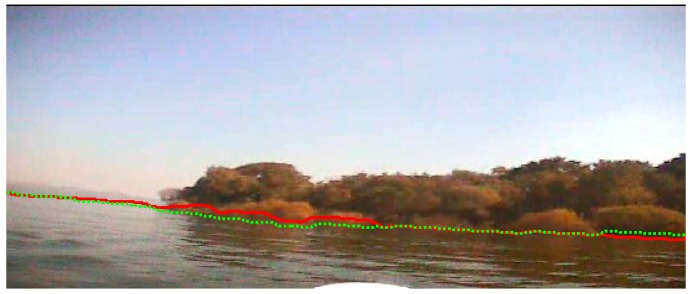
Precision validation of our method. The green dot line is the ground truth; the red solid line is the detection result of our method in this paper.

Then, we calculate the precision of our method. In the experiment, we detect the waterline of Figure 19a with our waterline detection method proposed in this paper, and compare it with the true ground which is obtained by manual pixel by pixel detection in the original image. The result is shown in Figure 22, where the red solid line is the detection result of our waterline detection method in this paper, and the green dot line is the ground truth which is obtained by manual detection. From Figure 22, we can see that the detected waterline is close to the true waterline. Then, we calculate the average difference between the detected waterline and the true waterline with Equation (15) and the mean square deviation with Equation (16) between them. The average difference is 1.84 pixels; The maximal difference is 6 pixels, and the minimal difference is 0 pixel. The mean square deviation is 4.57 pixels. The calculation formula of the average difference is denoted as:

(15) $$E_{1}=\frac{1}{n}\sum_{k=1}^{n}\left(H_{k}-{\tilde{H}}_{k}\right)$$

(16) $$E_{2}=\sqrt{\frac{1}{n}\sum_{k=1}^{n}{\left(H_{k}-{\tilde{H}}_{k}\right)}^{2}}$$

where *n* is the number of the sample points on the waterline; *H_k_* is the true coordinate of the waterline from manual detection, ${\tilde{H}}_{k}$ is the coordinate of the estimated waterline at the *k*th point from our detection method in this paper.

**Figure 23:**
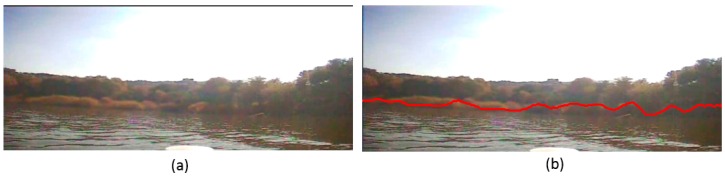
Waterline detection result when illumination is bad. (**a**) The image before our detection; (**b**) The image after our detection.

However, when the illumination conditions are so bad that both the trees and the shade of the trees on the bank are dark, it is difficult to judge where the waterline lies, and the detected waterline of our method in this paper has some errors at some positions. The experimental result can be seen in Figure 23. Therefore, in the future work, we will research deeply to improve the performance of our waterline detection method.

### 4.3. Comparison with Other Methods

**Figure 24:**
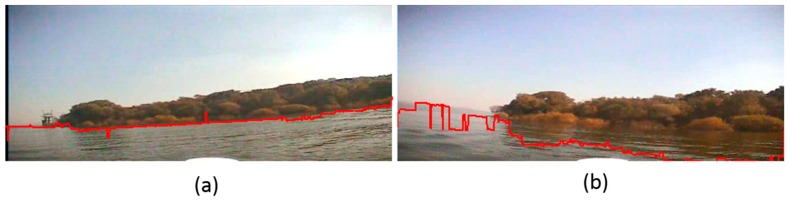
Waterline detection with current edge detection using the same images as Figure 17a and Figure 19a used to test our method in this paper. (**a**) The waterline detection using the same image as Figure 17a; (**b**) The waterline detection using the same image as Figure 19a.

In order to prove the precision of our waterline detection method in this paper, we now compare the processing result of other current methods with respect to the same sample images. First, we use edge detection combined with threshold segmentation to detect the waterlines of Figure 17a and Figure 19a, and the edge detection operator used is the canny operator. The detection result is shown in Figure 24, where we can see that for the image without shade, the detected waterline of current edge detection is close to that of our method, but there are some error points on the final waterline; while when a shade is projected on the water surface, the detected waterline of the edge detection method is far from the true ground. The reason is that the shade influences the intensity distribution of the water surface, and current edge detection which focuses on the variation of intensity cannot recognize the waterline in a shaded area.

**Figure 25:**
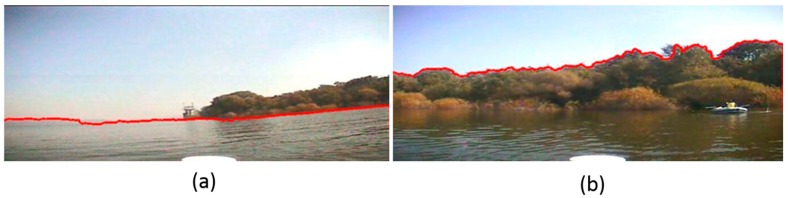
Waterline detection with super pixel segmentation using the same images as Figure 16a and Figure 20a used to test our method in this paper. (**a**) The waterline detection using the same image as Figure 16a; (**b**) The waterline detection using the same image as Figure 20a.

Then, we detect the waterline with the super pixel segmentation method, which refers to the digital image segmentation region for multiple image sequence process. Adjacent pixels have a series of position and features such as color, brightness, texture similar to that of small region composed of pixels. Most of the small area retains the further effective information for image segmentation, and generally does not destroy the boundary of the object in the image information. The images we use are Figure 16a and Figure 20a, and the result is shown as Figure 25, where we can see that when the illumination is good, the detection result with super pixel segmentation is close to that of our method in this paper, however, it cannot precisely segment the mountain whose color is close to that of the sky; While when the illumination condition is bad, the detection result with super pixel segmentation is totally wrong. Therefore, the precision of super pixel segmentation is sensitive to the illumination condition.

## 5. Conclusions

In this paper, we present an efficient waterline detection based on structure extraction and texture analysis with optical images, which was validated through practical application in a USV system. The primary contribution is introducing the basic method of structure extraction to preprocess river images so that the main structure and textures of the images remain, while the textures resulting from motion, wind, and illumination are removed. Then, GLCM and LBPs are combined, and the proposed method detects the waterline based on texture analysis. In the validation, the waterline of many images captured by our USV system outdoors was detected using the proposed detection method, and the results were compared with some other current waterline detection methods. The results showed that, compared to current methods based on edge detection and super pixel segmentation, the proposed algorithm is an effective and robust method for detecting complicated waterlines in real applications. The average error of the proposed method was 1.84 pixels, and the mean square deviation is 4.57 pixels. Our method can therefore potentially be used for USV navigation and control and ensuring the safety of the USV.

## Figures and Tables

**Table 1:** Parameters of the USV in our experiment.

Length	Width	Height	Max Velocity	Payload
2800 mm	700 mm	370 mm	35 km/h	70 kg

**Table 2:** Specifications of the VS250DH vision sensor.

Specification	Value
Version	vs-250DH
Focal length	5–50 mm
	(20 mm in this paper)
Aperature	F1.6
Imaging plane size	1/3"
Resolution	704 × 288 pixels
Minimal object distance	0.3 m
Minimal field of view	0.51 m
Angle of view	49.3°

**Table 3:** Experimental conditions.

Exp. ID	Riverbank Distance	Sunlight	Shade
I	far	even	none
II	closer	uneven	little
III	much coloser	uneven	little
IV	closest	uneven	heavy
